# Interventional treatment for neuropathic pain due to combined cervical radiculopathy and carpal tunnel syndrome: a case report

**DOI:** 10.1002/ccr3.840

**Published:** 2017-02-23

**Authors:** Simone Vigneri, Gianfranco Sindaco, Matteo Zanella, Elisabetta Sette, Valeria Tugnoli, Gilberto Pari

**Affiliations:** ^1^ Department of Experimental Biomedicine and Clinical Neurosciences (BioNeC)University of PalermoPalermoItaly; ^2^ Santa Maria Maddalena Hospital and Advanced Algology ResearchOcchiobelloItaly; ^3^ Department of Neuroscience/RehabilitationNeurophysiology UnitArcispedale Sant'AnnaUniversity of FerraraFerraraItaly

**Keywords:** Carpal tunnel syndrome, dorsal root ganglion, double‐crush syndrome, epidural treatment, pulsed radiofrequency

## Abstract

The coexistence of median and cervical nerve root damage might hide a complex pathophysiology. Here, we describe and discuss the case of a patient suffering from numbness and painful tingling of the hand, whose symptoms were effectively treated with pulsed radiofrequency and epidural administration of bupivacaine and morphine.

## Introduction

Carpal tunnel syndrome (CTS) occurs following the compression of median nerve, due to its passage through the flexor retinaculum at the wrist. Symptoms involve numbness and painful tingling of palm and first three fingers or even weakness of the affected hand, although diagnosis may become more challenging if symptoms extend proximally to the arm or sensitization signs occur 1. Moreover, the clinical picture may be complicated by the overlapping presence of peripheral neuropathy or cervical radiculopathy, worsening the outcome of common treatments 2. The failure of decompression surgery might be subsequent to incorrect diagnosis or incomplete release of the transverse carpal ligament promoting persistent symptoms, whereas recurrent symptoms seem to be related to fibrous proliferation and subtle palmar subluxation of the median nerve 3.

This report assesses whether some cases of CTS with poor response to treatments might actually be misdiagnosed due to a different pathophysiology, therefore requiring different therapeutical approaches. Moreover, this clinical case evaluates how the development of interventional treatments such as pulsed radiofrequency or epidural administration of drugs may promote symptom improvement in patients with complicated CTS.

## Case History

**Figure 1:**
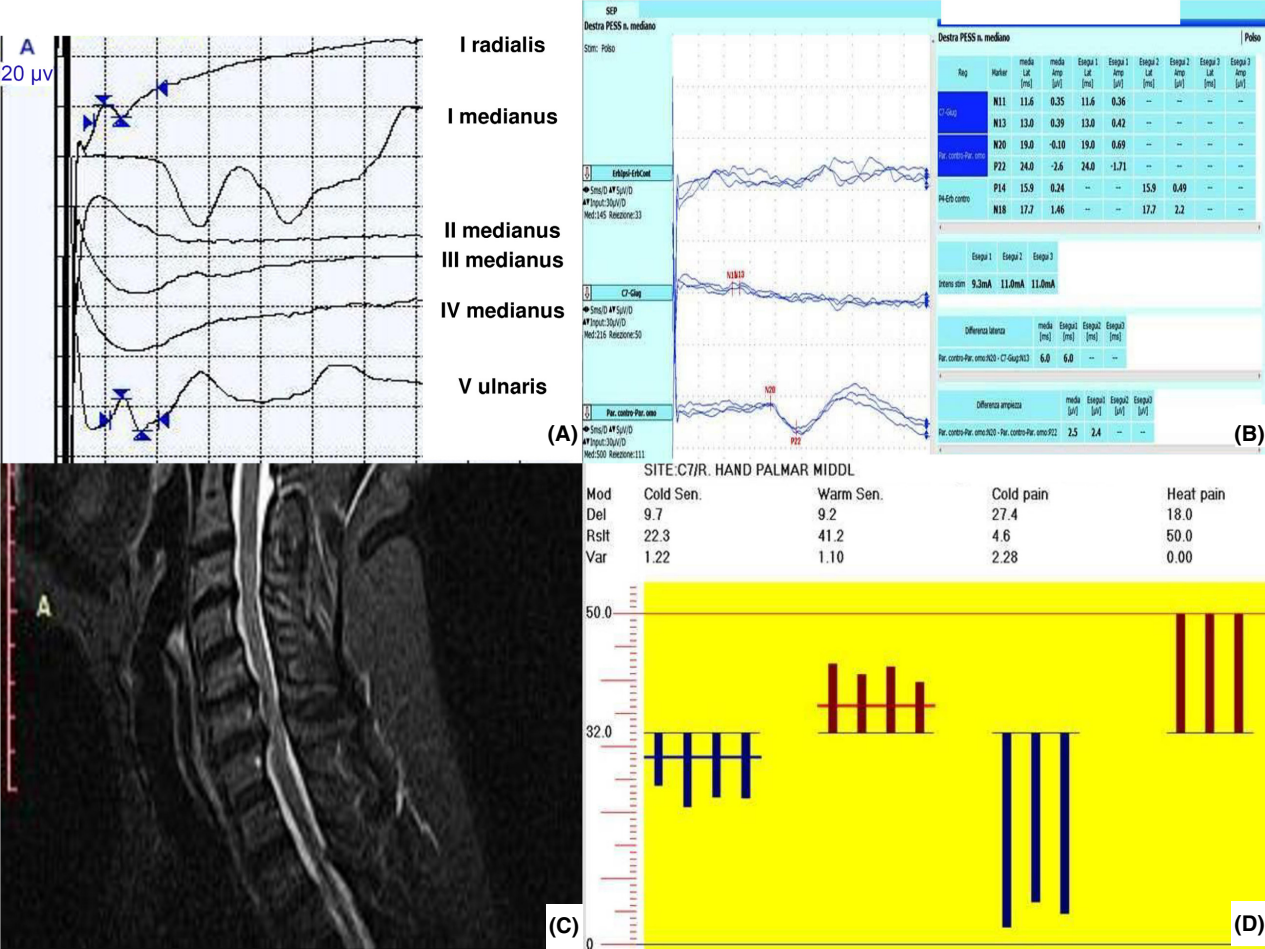
Imaging and neurophysiological tests. (A) Nerve conduction velocities; (B) median nerve somatosensory‐evoked potentials; (C) cervical MRI; (D) quantitative sensory testing.

A 69‐year‐old woman with unremarkable medical history was suffering for one year from progressive painful tingling sensation in the fingertips of her right hand. Most common pharmacological treatments such as pregabalin and amytriptiline were used with no symptom relief. Nerve conduction velocity (NCV) test could not detect any sensory potential from the median nerve, whereas remaining nerve conductions were preserved in the right hand (Fig. 1A), thereby suggesting an underlying CTS with no significant relief of symptoms after surgical treatment.

She was admitted due to severe worsening of pain (NRS 8, DN4 4/10) in the palm and first three fingers of the right hand although follow‐up ruled out complications due to surgery. Symptoms were reported as continuous numbness and tingling worsened by electric shock sensations evoked by touch and hand movements. Pain was not related to posture nor radiating to another site on the arm. Clinical examination showed mild weakness of the hand with pinprick hypoesthesia and dynamic tactile allodynia in the palm. Sensory‐evoked potentials from the right median nerve confirmed an impaired somatosensory conduction of peripheral origin (Fig. 1B). Right median nerve F‐wave latency and ulnar NCV were normal (Table 1) but needle examination displayed a chronic radicular involvement of C6‐C7 and C7‐C8 levels on the right side. MRI did not show any signal intensity variation nor edema of the right median nerve, whereas cervical radiculopathy was displayed at the right C5‐C6 and C6‐C7 root level (Fig. 1C). Last, quantitative sensory testing highlighted thermal and pain hypoesthesia mainly affecting warm sensation restricted to the painful territory (Fig. 1D). Blood tests were negative for metabolic or inflammatory disorders.

**Table 1:** Nerve conduction velocity values and F‐responses from the right hand

	DL/MFL (msec)	Amp (*μ*V/mV)	Vel (msec)
Right radialis SAP (I finger)	1.5	4.3	50.1
Right medianus SAP (II, III, and IV finger)	u.p	u.p	u.p.
Right ulnaris SAP (V finger)	1.9	13.1	49.4
Right medianus cMAP (distal)	3.7	6.3	–
Right medianus cMAP (proximal)	9.0	7.6	52.5
Right ulnaris cMAP (distal)	2.0	12.4	–
Right ulnaris cMAP (proximal)	6.0	10.2	57.5
Right medianus F‐response	23.9	–	–

DL/MFL, distal latency/minimal F latency; Amp, amplitude; Vel, velocity; SAP, sensory action potential; cMAP, compound motor action potential; u.p., unelicitable potential.

**Figure 2:**
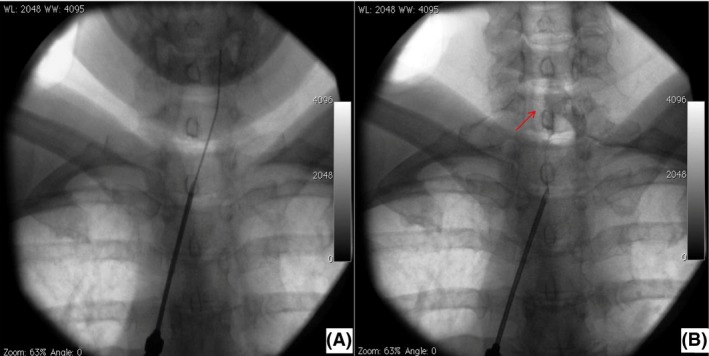
(A) Pulsed radiofrequency treatment of the cervical dorsal root ganglion. (B) Placement of epidural catheter and spread of X‐ray contrast medium (arrow).

A local injection of steroids at the wrist was ineffective and ruled out a mere distal nerve entrapment, whereas subsequent ultrasound‐guided radicular transforaminal administration of steroids and lidocaine resulted in immediate pain relief. Considering the trial effectiveness, we enrolled the patient for pulsed radiofrequency of C5‐C6‐C7 dorsal root ganglion (DRG) and the insertion of a cervical epidural catheter for pharmacological treatment 4, 5. The procedure was performed in a safe and quiet operating room. The patient was lying in a prone position, and after the injection of local anesthetic, a 16‐G hollow needle was introduced into the T1‐T2 interspace and epidural space located by loss of resistance to air technique. A pulsed radiofrequency electrode (Reig‐Cosman Electrode) was introduced through the needle and moved with its active tip into the cervical epidural space close to the right DRG at C5 level. Proper placement of the catheter was confirmed with fluoroscopic projections. After connecting the probe to a generator (Cosman G4), sensory (50‐Hz) and motor (2‐Hz) stimulation tests were performed in order to confirm the correct electrode position with no motor recruitment. Pulsed radiofrequency (2 Hz) was applied for 240 sec at 45 V with a tip temperature between 40 and 42°C. Prior to the electrode removal, the procedure was repeated with the same technique on the right DRG at C6 and C7 levels (Fig. 2A). An epidural catheter was subsequently inserted and moved up to the target area (Fig. 2B). Subarachnoid or intravascular placement of the catheter was ruled out by the injection of X‐ray contrast medium (Iopamiro 300, Bracco Imaging Italia srl). The catheter was tunneled for 5 cm subcutaneously and then fixed on the skin.

The epidural solution was made up with morphine chlorhydrate (0.1 mg/mL) and bupivacaine (1 mg/mL), and the medication (3 mL three times/day) was administered during 4 weeks prior to the catheter removal. No complications resulted from the treatment. Outcome measures were obtained at 3 months postprocedure with clinical examination and NRS. Tactile allodynia faded away few minutes after the intraoperative first infusion, whereas significant improvement of paresthesia and pain (NRS 2) but poor effectiveness on hypoesthesia was reported by the patient at follow‐up.

## Discussion

In our patient, symptoms and pain distribution were resembling a carpal tunnel syndrome. Palmar cutaneous branch damage after unsuccessful median nerve release at the wrist cannot be ruled out. Nevertheless, neurophysiological tests disclosed median nerve large‐ and small‐fiber (mainly affecting C fibers) neuropathy and peripheral somatosensory conduction slowing in the affected upper limb. Moreover, the poor response to local drug injections and surgery, the rapid and long‐lasting pain relief obtained following PRF and epidural drug administration raised up the hypothesis of a cervical proximal damage which was confirmed by imaging.

Although we cannot determine which lesion occurred first, clinical signs and symptoms might be subsequent to the coexistence of cervical radiculopathy and distal entrapment neuropathy, often described with the term “double‐crush syndrome” (DCS) 6, 7. DCS was proposed to describe two focal lesions along the same axon where the proximal one increases the chance of damage in the distal site. This controversial diagnosis has been widely debated because its symptoms may hardly arise due to impaired axoplasmic flow, due to separate sets of microtubules and axonal transport systems between proximal and distal branches. Nevertheless, different hypotheses have been postulated. For instance, sensory action potentials might be directly transmitted from the periphery to the central processes, bypassing cell bodies. Lymphatic or venous drainage impairment and endoneurial edema at proximal site might subsequently affect the nerve distally 8.

Epidural delivery of anesthetics has been associated with long‐lasting improvement of pain and mechanical allodynia in animals and humans. Ongoing discharges in hyperexcitable DRG neurons after nerve injury might induce central sensitization and tactile allodynia, which can be reversed without nerve conduction impairment by the injection of systemic lidocaine 9. In a previous study performed in patients with phantom limb pain, the administration of lidocaine intrathecally and to the DRG surface promoted transient improvement of painful and nonpainful sensations in most of patients, likely due to the suppression of spontaneous bursting discharge and sensitization 10. Although recommendations for PRF are mostly inconclusive in radicular pain, its use in cervical radicular pain may be indicated for a selected group of patients 11. Moreover, a rising number of scientific works with PRF is focusing on strict enrollment criteria (e.g., nociceptive vs. neuropathic pain) and stimulation parameters 12. The electrical stimulation of cervical DRG was also reported to promote significant pain relief in a patient with complex regional pain syndrome. The recruitment of large‐ and small‐fiber neurons in the DRG might be responsible for improvement of pain, allodynia, and skin perfusion 13. Therefore, the combined effects of pulsed radiofrequency and epidural anesthetic might exert their effects by restoring the physiological excitability of the targeted DRG.

## Conclusions

Regardless of its pathophysiology, the significant improvement in our patient might explain the therapeutic failure of several misdiagnosed cases of CTS. Therefore, clinical symptoms suggestive of distal entrapment neuropathy in the upper limb might actually conceal a different pathogenesis and, if associated with cervical involvement, might be significantly relieved by combined pulsed radiofrequency and epidural medications.

## Authorship

SV: involved in conception and organization of research project; in review and critique of the data; and in writing of the first draft and in review and critique of the manuscript. GS: involved in organization and execution of research project and involved in review and critique of the manuscript. MZ: involved in organization and execution of research project. ES: involved in execution of research project and acquired the data. VT: involved in organization of research project; involved in review and critique of the data; and involved in review and critique of the manuscript. GP: involved in conception of research project and in review and critique of the manuscript.

## Consent

Written informed consent was obtained from the patient for publication of this case report. A copy of the written consent is available for review by the Series Editor of this journal.

## Conflict of Interest

The authors declare no conflict of interest.
